# Formation of Three-Way Scanning Electron Microscope Moiré on Micro/Nanostructures

**DOI:** 10.1155/2014/281954

**Published:** 2014-02-11

**Authors:** Qinghua Wang, Satoshi Kishimoto, Hiroshi Tsuda

**Affiliations:** ^1^Research Institute of Instrumentation Frontier, National Institute of Advanced Industrial Science and Technology, 1-1-1 Umezono, Tsukuba, Ibaraki 305-8568, Japan; ^2^Hybrid Materials Unit, National Institute for Materials Science, 1-2-1 Sengen, Tsukuba, Ibaraki 305-0047, Japan

## Abstract

Three-way scanning electron microscope (SEM) moiré was first generated using a designed three-way electron beam (EB) in an SEM. The spot-type three-way SEM moiré comes from the interference between the three-way EB and the specimen grating in which the periodic cells are arranged in a triangular manner. The deformation and the structure information of the specimen grating in three directions can be simultaneously obtained from the three-way SEM moiré. The design considerations of the three-way EB were discussed. As an illustration, the three-way SEM moiré spots produced on a silicon slide were presented. The proposed three-way SEM moiré method is expected to characterize micro/nanostructures in triangular or hexagonal arrangements in three directions at the same time.

## 1. Introduction

Among various micro/nanooptical measurement techniques, the moiré method [1] has been developed into a mature deformation measurement technique due to its noncontact and full-field properties. In the middle of 1980s, moiré interferometry [2, 3] was proposed and gradually improved to study the mechanical behaviors of materials. With the development of high-resolution microscopes, the micro/nanomoiré methods have got fast development and wide applications. The scanning lines of microscopes instead of actual gratings can be regarded as reference gratings, and the implements of high-resolution microscope moiré methods are fairly simple. Since the electron beam (EB) moiré or scanning electronic microscope (SEM) moiré method was firstly put forward [4, 5], this method has been widely used for micro/nanodeformation measurement and the further stress analysis [6–9].

Furthermore, many other high-resolution microscope moiré methods have been proposed and developed, such as the focused ion beam (FIB) moiré method [10], the atomic force microscope (AFM) moiré method [11], the scanning tunneling microscope (STM) moiré method [12], the transmission electron microscope (TEM) moiré method [13], and the laser scanning confocal microscope (LSCM) moiré method [14]. In addition, the digital micro/nanomoiré method using a digital grating as the reference grating has also been applied to measure micro/nanodeformation [15, 16]. Besides deformation measurement, micro/nanostructure characterization can also be realized from micro/nanomoiré fringes, such as the LSCM moiré fringes [17], the SEM moiré fringes [18], the secondary moiré fringes [19], and the three-way digital moiré fringes [20, 21].

Each moiré method has its own advantages and disadvantages. Considering that numerous micro/nanostructures are arranged in triangular manners, we first generate three-way SEM moiré fringes in this study to analyze the deformation as well as the structure information in three directions at the same time.

## 2. Formation and Measurement Principles of Three-Way SEM Moiré

### 2.1. Formation Principle of One-Way SEM Moiré

**Figure 1:**
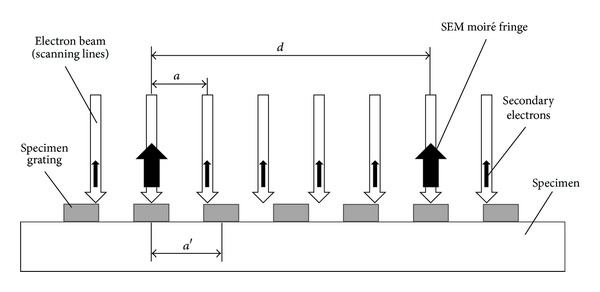
Formation principle of one-way SEM moiré fringes.

In the traditional SEM moiré method, SEM moiré fringes come from the interference between the scanning lines of a SEM and the specimen grating, as shown in Figure 1. The specimen grating (also called model grid) can be found from the natural periodic structure of the sample or be fabricated on the sample surface by various methods such as photolithography. The scanning lines of the SEM which have almost the same pitch (spacing) as that of the specimen grating can be used as the reference grating (also called master grid). If there is a mismatch or misalignment between the SEM scanning lines and the specimen grating, the difference in the amount of emitted secondary electrons per primary electron will produce the SEM moiré fringes which consists of periodically distributed bright and dark parts [22]. Because the scanning lines are in the same direction, the formed moiré fringes are one-way SEM moiré fringes. Using the traditional SEM moiré fringes, the deformation and the structure information of a sample in the direction perpendicular to the scanning lines can be analyzed.

### 2.2. Formation Principle of Three-Way SEM Moiré

**Figure 2:**
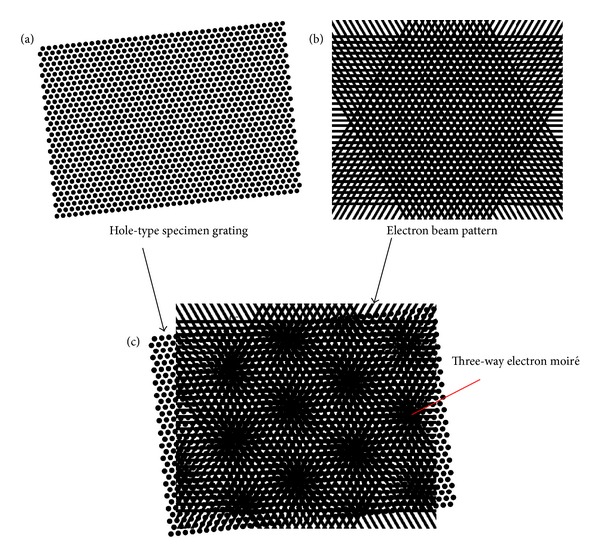
Formation principle of three-way SEM moiré for a hole-type specimen grating. In (b), the white part means the exposed EB and the black part expresses the unexposed EB.

When the specimen grating is a three-way grating; that is, the periodic structure on the sample surface is arranged in a triangular manner, we can design an EB pattern containing three groups of scanning lines in three directions. If the specimen grating is hole-type such as a nanoporous structure; that is, the periodic dots or cells are holes, the SEM image of the specimen grating will be similar to the schematic diagram in Figure 2(a) in which the periodic dots are black due to less secondary electrons. In this case we will design an EB pattern as illustrated in Figure 2(b), in which the periodic white dots represent the exposed EB and the black lines express the unexposed EB. The interference between the specimen grating in Figure 2(a) and the EB in Figure 2(b) will generate the three-way electron moiré as shown in Figure 2(c).

**Figure 3:**
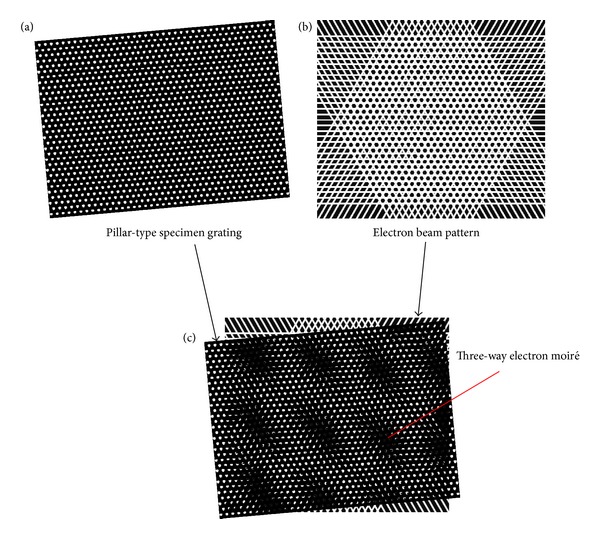
Formation principle of three-way SEM moiré for a pillar-type specimen grating. In (b), the white part denotes the exposed EB and the black part represents the unexposed EB.

For the pillar-type specimen grating such as a nanoparticle structure; that is, the periodic dots are pillars, the SEM image of the specimen grating will be similar to Figure 3(a) in which the periodic dots are white owing to more secondary electrons. Then, we will design an EB pattern presented in Figure 3(b), in which the periodic black dots stand for the unexposed EB and the white lines denote the exposed EB. When the EB in Figure 3(b) is superimposed on the specimen grating in Figure 3(a), the spot-type three-way SEM moiré will emerge as shown in Figure 3(c).

Figure 3(b) looks like the inverted gray scale image of Figure 2(b). If we use Figure 3(b) to interference with Figure 2(a), or use Figure 2(b) to interfere with Figure 3(a), we can get hexagonal moiré fringes [20] which look like the inverted gray scale pattern of Figures 2(c) or 3(c). As the spot-type moiré is more distinct than the hexagonal moiré, we choose to form the spot-type moiré in Figures 2(c) and 3(c) as the three-way SEM moiré.

The difference between the spot shapes of the three-way SEM moiré in Figures 2(c) and 3(c) suggests the different deformation of the specimen grating relative to the three-way EB. If the deformation of the specimen grating relative to the EB can be expressed by uniform rotation and uniform expansion or shrink, the SEM moiré spots will be in a circular type as seen from Figure 2(c). If the deformation of the specimen grating relative to the EB is not uniform in three directions, the SEM moiré spots will be close to an elliptical shape as displayed in Figure 3(c).

### 2.3. Measurement Principle of Three-Way SEM Moiré Method

**Figure 4:**
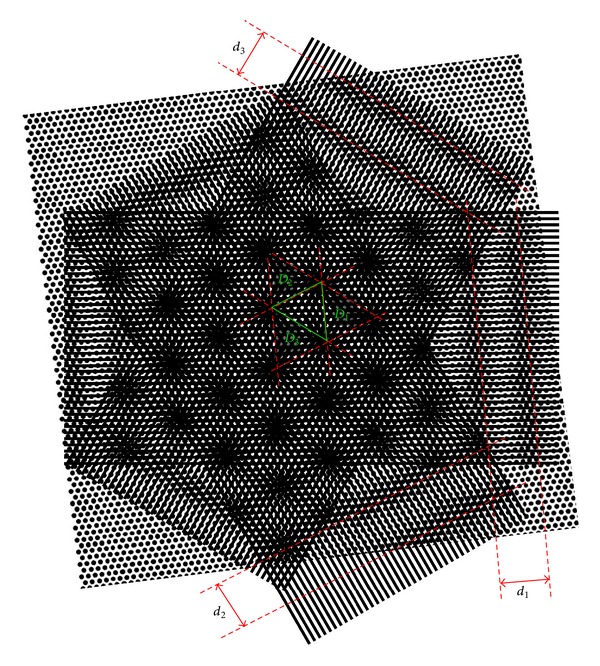
Relationship between three-way SEM moiré and one-way SEM moiré.

Figure 4 shows the relationship between the three-way SEM moiré and the traditional one-way SEM moiré. If we divide the three-way EBs into three one-way EBs, we can find three groups of one-way SEM moiré fringes which are, respectively, generated from the interferences between the three one-way EBs and the specimen grating. From Figure 4, the three-way SEM moiré spots result from the interferences among the three groups of one-way SEM moiré fringes.

Let *D*_1_, *D*_2_, and *D*_3_ represent the distances between each two spot centers of three adjacent SEM moiré spots, respectively, and *d*_1_, *d*_2_, and *d*_3_ mean the fringe spacings of three groups of one-way SEM moiré fringes, respectively. The quantitative relationship between *D*_1_, *D*_2_, *D*_3_ and *d*_1_, *d*_2_, *d*_3_ can be obtained based on the geometric relationship of a triangle labelled in Figure 4. *D*_1_, *D*_2_, and *D*_3_ are the three side lengths and *d*_1_, *d*_2_, and *d*_3_ signify the three altitudes of the triangle. The variables of *d*_1_, *d*_2_, *d*_3_ can be calculated by

(1) di=(D1+D2+D3)(D1+D2−D3)(D2+D3−D1)(D3+D1−D2)2Di                           (i=1,2,3).

As a consequence, after *D*_1_, *D*_2_, and *D*_3_ are measured from the three-way SEM moiré image, we can easily use the tradition SEM moiré method to get the deformation and the structure information of the specimen grating which are related to *d*_1_, *d*_2_, and *d*_3_. The calculation formula of the deformation of the specimen grating relative to the EB in the traditional SEM moiré method can be found in [22, equation (1)]. The calculation formulas of the pitch and the orientation angle of the specimen grating in three directions can be seen in [20, equation (2)].

An obvious advantage of the three-way SEM moiré method is that the deformation and the structure information of the micro/nanospecimen grating in three directions can be simultaneously obtained. Using the three-way EB to replace the three-way digital grating [20] as the reference grating is expected to reach a wider view field.

## 3. Design of the Three-Way EB

In the implementation process of the three-way SEM moiré method, design of an EB pattern is a crucial step. There are three aspects that need to be considered.

**Figure 5:**
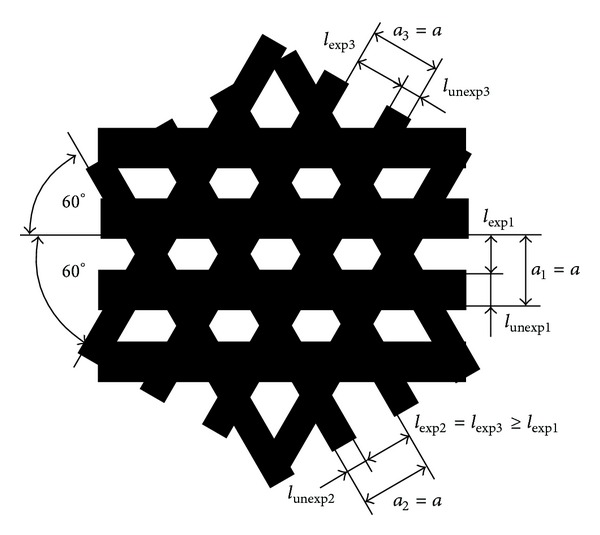
Size design of the three-way EB pattern.

**Figure 6:**
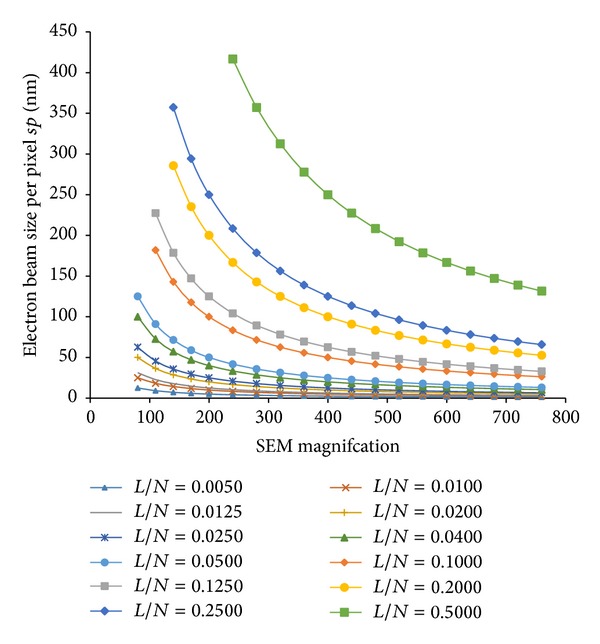
Variation of the EB size per pixel (*sp*) along with the SEM magnification.

(1) It is better to use the EB pattern in Figure 2(b) for a hole-type specimen grating and in Figure 3(b) for a pillar-type specimen grating. We can introduce Figure 2(b) into a SEM, and if we choose “Reverse” in the process of scanning, the used EB pattern is almost the same as Figure 3(b).
(2) Due to the exposure lag of the EB in the scanning process, the real exposed region is smaller than the designed exposed region if there are both exposed and unexposed regions in an EB pattern. Consequently, it is recommended that the exposed sizes of the oblique one-way lines (*l*_exp2_ and *l*_exp3_ in Figure 5) should be equal to or greater than that of the horizontal one-way line (*l*_exp1_ in Figure 5) of the designed three-way EB pattern. Usually, *l*_exp2_ = *l*_exp3_ is 1~1.2 times *l*_exp1_. To facilitate calculation, the pitch (*a*_*i*_), that is, the sum of the exposed size (*l*_exp⁡*i*_) and the unexposed size (*l*_unexp*i*_) of each one-way EB line, is better to be set to be equal, where *i* = 1, 2, 3. So we can use one symbol *a* (*a* = *a*_*i*_) to express the pitch of the three-way EB. Besides, the included angles between each two one-way lines of the three-way EB is usually set to be 60° (Figure 5) for easy calculation.
(3) To form three-way SEM moiré, the pitch (*a*) of the three-way EB should be close to the specimen grating pitch. Usually, *a* is 0.8~1.2 times the pitches (*a*_*i*_′) of the specimen grating in three directions, where *i* = 1, 2, 3. Then, we will discuss how to control the EB pitch when using the SPG-724 pattern generator in an SEM. In this pattern generator, only a square region can be exposed. The pixel number (*N*) and the length (*L*) of this square region under the SEM magnification of 200x can be chosen from some options. The options include *N* = 1000, 2000, 4000, 5000, and 10000 pixels and *L* = 50, 100, 200, and 500 *μ*m. If we choose different values of  *N*,  *L*  and different SEM magnifications, the EB sizes will be different. The variation of the EB size in nanometer per pixel along with the SEM magnification under different combinations of *N* and *L* is plotted in Figure 6. The EB pitch can be calculated by
  (2) $$\begin{array}{c} a=n*sp, \end{array}$$

where *n* is the pixel number of the EB pitch and *sp* represents the EB size in nanometer per pixel which can be determined from Figure 6.

## 4. Observation of Three-Way SEM Moiré

In this section, we will show the observed three-way SEM moiré taking a hole-type specimen grating (grid) as an example. The specimen grating in which the holes are arranged in a triangular manner was produced by the EB lithography (EBL) method [5]. The used SEM (SX-40A) is equipped with a pattern generator (SPG-724).

**Figure 7:**
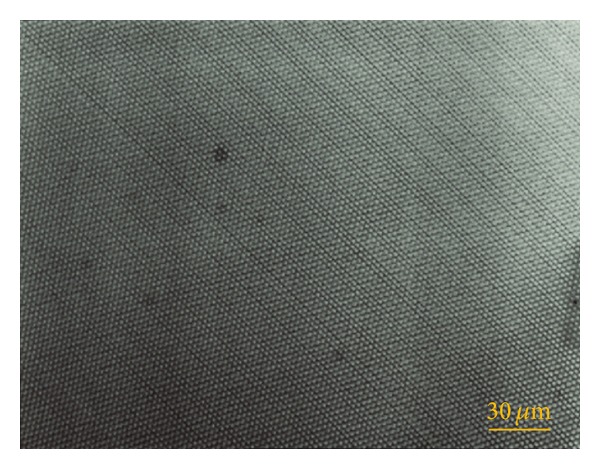
The hole-type three-way grating on silicon fabricated by the EBL method.

The procedure for fabricating the grid includes four steps. First, a silicon slide was covered with an electron-sensitive layer (EB resist, EBR9) by a spin coater. Then, an EB pattern was designed according to the desired grating shape and size. Next, the silicon slide was placed on the specimen stage in the SEM and the EB pattern was introduced for the EB exposure. Finally, the silicon slide with the EB resist was developed and then immediately rinsed. When the exposed EB resist was removed, the three-way grating pattern with a pitch of about 2.05 *μ*m emerged on the specimen.  Figure 7 exhibits the specimen grating image recorded by a Lasertec scanning laser microscope (1LM15). It should be noted that although we can design an EB pattern in which the unexposed areas are distributed in arbitrary shapes and regions, the EB scans row by row in the horizontal direction. The reason why one direction is more distinct than the other two directions is that the distinct direction is in the horizontal direction during the EBL process.

Before generating three-way SEM moiré on this silicon slide, we should determine the used EB pattern. For this hole-type specimen grating, we adopted the EB pattern similar to Figure 2(b). Since the specimen grating pitch is about 2.05 *μ*m, the designed EB pitch should be in the range of 1.64–2.46 *μ*m (0.8–1.2 times). Next, suitable options should be chosen to realize an applicable EB pitch in the SEM and the pattern generator which were the same as in the EBL process. The pixel number of the EB pitch was set to be *n* = 50, with the exposed pixels of 20 and the unexposed pixels of 30. The pixel number and the length of a square in the pattern generator were chosen to be *N* = 5000 pixels, an *L* = 500 *μ*m from the existing options. When scanning, the magnification of the SEM was chosen as 500x. From Figure 6, it is found that the EB size per pixel is *sp* = 40 nm in the case of  *L*/*N* = 0.1. Therefore, the EB pitch can be calculated using (2); that is, *a* = 2 *μ*m.

**Figure 8:**
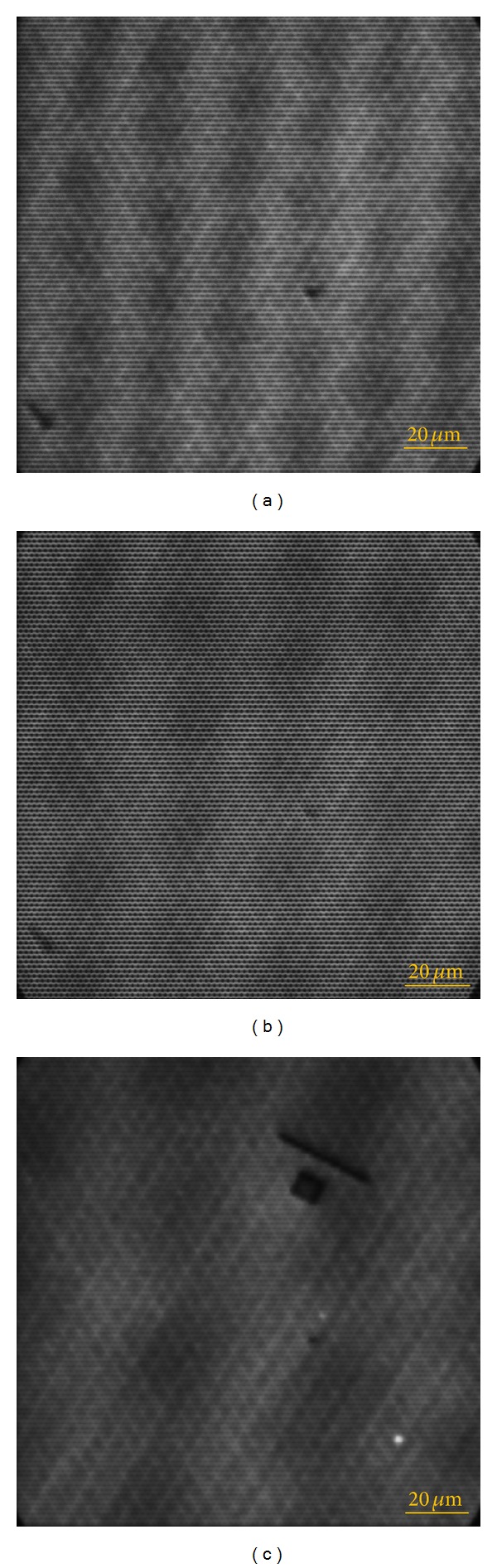
The three-way SEM moiré patterns on silicon. (a) The moiré spots are not distinct, (b) when the EB pattern is clockwise rotated by 1°, and (c) when the EB pattern is clockwise rotated by 2°.

When the designed EB pattern is used to scan on the specimen grating on the silicon slide, the “Spot scan” mode in the SPG-724 pattern generator should be adopted. The exposure dose and the electric current are better to be small. In this experiment, the exposure dose was 1 *μ*C/cm^2^ and the electric current was 70 pA measured by a digital electrometer (8252, ADCMT). When the EB patterns are at an appropriate position, three-way SEM moiré will appear (Figure 8(a)). But the three-way SEM moiré is not distinct in Figure 8(a). In this case we can rotate the EB slightly until distinct three-way SEM moiré emerges. Figures 8(b) and 8(c) show the three-way SEM moire patterns when the EB used in Figure 8(a) is clockwise rotated by 1° and 2°, respectively. Both the three-way SEM moiré patterns in Figures 8(a) and 8(b) are clear, and can be used to characterize the specimen grating on silicon in three directions at the same time, based on the measurement principle of the three-way SEM moiré method mentioned in Section 2.3.

## 5. Conclusions

The three-way SEM moiré method was proposed for deformation and structure analysis of periodic structures in triangular or hexagonal arrangements. The three-way SEM moiré is derived from the interference between the three-way EB and the specimen grating. It can also be regarded as the result of the interference among the three groups of one-way SEM moiré fringes. The measurement principle for the deformation and the structure pitch as well as the orientation from the three-way SEM moiré pattern was presented. Three key points of design about the three-way EB were pointed out. The operation sequence and the matters needing attention in the forming process of the three-way SEM moiré were demonstrated taking a hole-type specimen grating on silicon as the example. The three-way SEM moiré method is able to simultaneously characterize micro/nanostructures in three directions. This method is promising in determining the domain boundary or grain boundary of micro/nanostructures from the difference of moiré spots in different domains or grains in a large view field.
